# Socio-demographic factors associated with pet ownership amongst adolescents from a UK birth cohort

**DOI:** 10.1186/s12917-019-2063-x

**Published:** 2019-09-18

**Authors:** Rebecca Purewal, Robert Christley, Katarzyna Kordas, Carol Joinson, Kerstin Meints, Nancy Gee, Carri Westgarth

**Affiliations:** 1 Institute of Infection and Global Health, and Institute of Veterinary Science, Faculty of Health and Life Sciences, University of Liverpool, Leahurst Campus, Neston, Cheshire CH64 7TE UK; 2 Department of Epidemiology and Environmental Health, University at Buffalo, 270 Farber Hall, Buffalo, NY 14214 USA; 3 School of Social and Community Medicine, University of Bristol, 39 Whatley Road, Bristol, BS8 2PS UK; 4 School of Psychology, University of Lincoln, Sarah Swift Building, Brayford Wharf East, Lincoln, Lincolnshire LN5 7AY UK; 5 Department of Psychology, State University of New York, Fredonia, NY 14063 USA; 6 WALTHAM Centre for Pet Nutrition, Waltham-on-the-Wolds, Leics, LE14 4RT UK

**Keywords:** ALSPAC, Pet ownership, Dog, Cat, Adolescent

## Abstract

**Background:**

In developed nations, pet ownership is common within families. Both physical and psychological health benefits may result from owning a pet during childhood and adolescence. However, it is difficult to determine whether these benefits are due to pet ownership directly or to factors linked to both pet ownership and health. Previous research found associations between a range of socio-demographic factors and pet ownership in seven-year-old children from a UK cohort. The current study extends this research to adolescence, considering that these factors may be important to consider in future Human-Animal Interaction (HAI) research across childhood.

**Results:**

The Avon Longitudinal Study of Parents and Children (ALSPAC) collected pet ownership data prospectively via maternal reports from gestation up to age 10 years old and via self-report retrospectively at age 18 for ages 11 (*n* = 3063) to 18 years old (*n* = 3098) on cats, dogs, rabbits, rodents, birds, fish, tortoise/turtles and horses. The dataset also contains a wide range of potential confounders, including demographic and socio-economic variables. The ownership of all pet types peaked at age 11 (80%) and then decreased during adolescence, with the exclusion of cats which remained constant (around 30%), and dogs which increased through 11–18 years (26–37%). Logistic regression was used to build multivariable models for ownership of each pet type at age 13 years, and the factors identified in these models were compared to previously published data for 7 year-olds in the same cohort. There was some consistency with predictors reported at age 7. Generally sex, birth order, maternal age, maternal education, number of people in the household, house type, and concurrent ownership of other pets were associated with pet ownership at both 7 and 13 years (the direction of association varied according to pet type). Factors that were no longer associated with adolescent pet ownership included child ethnicity, paternal education, and parental social class.

**Conclusions:**

A number of socio-demographic factors are associated with pet ownership in childhood and adolescence and they differ according to the type of pet, and age of child. These factors are potential confounders that must be considered in future HAI studies.

## Background

The study of Human-Animal Interactions (HAI) is an expanding field of research. HAI is the mutual and dynamic relationship between people and animals, and the effects these interactions have on physical and psychological health and well-being of both people and their pets [1]. Potential benefits of pet ownership on the emotional and physical health of both adults [2–10] and children [11–15] have been observed.

Pets may play a distinctive role in supporting well-being in adolescence because it is a developmental period characterized by a great deal of emotional and physical change due to sexual maturation. From a psychological health perspective, pet ownership in adolescence has been shown to enhance self-esteem [16–19], decrease loneliness [20–22], and increase resilience to depressive [22] and anxious symptoms [13]. However, the research is not conclusive; some studies have found null effects on these outcomes [23–25]. Pet ownership has also been associated with educational [26] and cognitive development [27] of youths. Dogs in particular have been found by some to improve physical activity [28, 29], although others report no benefit [30, 31].

Mixed findings may in part be due to methodological differences among studies [11]. The inconsistent evidence regarding the health impacts of pet ownership in adolescence is a common problem in HAI studies and may be due to a wide diversity of designs, small effect sizes, and small and homogeneous self-selected samples, as well as incomplete adjustment for relevant confounders [32]. Methodological limitations also reduce the ability to infer causality [11, 33]. Further research into the health effects of pet ownership during childhood and adolescence is required. The use of appropriate methodology, including adjustment for confounders, is critical to ensure findings are not over-interpreted, nor any tangible associations missed [33].

Socio-demographic factors may explain postulated psychological and physical health benefits of pets [11, 34–37]. Although many studies adjust for at least age and sex of the participants, pet ownership has been associated with other factors [37–40], such as ethnicity, the number of people in a household, the presence of an older sibling, parental education and social class, maternal age at delivery, maternal pet ownership history and housing type [37]. The need to control for confounding factors is recognised; studies have identified socio-demographic differences in ownership of different pets types in adults [34, 36, 41] and children [37, 42], but less so in adolescents [43, 44].

If we are to examine the evidence for health benefits of pet ownership in adolescence, we first need to understand the factors associated with pet ownership. We need to explore which socioeconomic, demographic and behavioural variables are associated with ownership of different pet types, so that they can be controlled for as much as possible during study design and analysis of data involving HAI. Differences between explanatory factors associated with ownership of different types of pets also need to be examined as differences in the type of people who own them have been found [37, 45] including; social class, education level, household composition, gender of respondents, and house type. Previous research is mostly limited to dog and cat ownership [34, 36, 38].

Birth cohorts are useful sources of data to examine factors associated with pet ownership, and have been used for this purpose in studies of children [37]. However, differences may exist in the prevalence and frequency of pet ownership among children and adolescents, and there may be differences in the variables that explain pet ownership in childhood and adolescence [37, 42, 46, 47]. Furthermore, because youth interaction with pets is mediated by interactions with adults, siblings, and peers, a life-course approach is needed to specify mediational models and pathways in human health outcomes over time [32, 48]. In addition, previous research shows conflicting associations for example, whether pet owners had higher [36] and lower [41] education levels than not pet-owners. The use of a very large sample in the present study provides advantage over previous research, due to the likelihood of being more representative.

Given the relative paucity of studies on the sociodemographics of pet ownership among adolescents [19, 44], the present study assesses which sociodemographic variables are important in determining pet ownership of different types of pets in a large UK birth cohort study.

The aim of this study was to use the Avon Longitudinal Study of Parents and Children (ALSPAC) to describe pet ownership during adolescence in terms of prevalence and predictors, and to compare to findings from the same cohort during childhood.

Objectives are as follows:

1. Describe the prevalence of the ownership of different pet types, and how these change throughout childhood and adolescence, from infancy up to age 18 years.
2. Identify and describe the potential confounding factors associated with ownership of each pet type in adolescence at age 13 years. This age was chosen for examination as it marks the beginning of adolescence and is a period of great change in terms of pubertal, cognitive and socio-emotional development. In addition, this age group was ideal in terms of sample size for each model; pet ownership of smaller pet types was expected to decrease in later adolescence.

## Method

The Avon Longitudinal Study of Parents and Children (ALSPAC) is a UK prospective birth cohort study that has been described in detail elsewhere [49]. Briefly, 14,541 pregnant women were recruited with expected delivery dates between 1st April 1991 and 31st December 1992. Of the 13,978 singletons/twins alive at 1 year, a small number of participants withdrew consent (*n* = 24) leaving a starting sample of 13,954. Data were collected from pregnancy onwards using postal questionnaires, clinic assessments, biological samples, linkage to routine information, abstraction from medical records and environmental monitoring. The study website contains a searchable dictionary of all the available data (http://www.bris.ac.uk/alspac/researchers/data-access/data-dictionary/). Ethical approval was obtained from the ALSPAC Law and Ethics Committee and the Local Research Ethics Committees; the participants provided written informed consent. As ethical approval and consent was sought as part of the data collection process for ALSPAC, and as this study analyses retrospective data, no ethical approval or consent was specifically required for the present study.

Pet ownership was reported by the mothers of 13,557 children during gestation, caregivers of 7800 children by age 10 years, and by 3098 adolescents at age 18 years for ages 11–18 years. The pet ownership data from gestation up to age 10 years has been previously analysed and described in detail [37]. In addition, age 7 pet ownership data were collected retrospectively to assess the accuracy of participants’ recall.

At each age, participants were asked to recall whether they had any pets in their household and if so, how many pets they had of each type. Pet type included cats, dogs, rabbits, rodents (mice, hamster, gerbil, etc.), birds (budgerigar, parrot, etc.), fish, tortoises/turtles and horses. Horse ownership had not been recorded in the childhood (0–10 years) pet ownership dataset.

### Data analysis

To enable the comparison of pet ownership history across childhood and adolescence in terms of ‘never’, ‘sometimes’ or ‘always’ owned pets, a two-step cluster method was repeated from the initial paper on childhood pet ownership [37] using the adolescent data. The two-step cluster method, carried out in SPSS version 24, categorised groups of children in the dataset according to their pet ownership history using a scalable cluster analysis algorithm. Children were organised into groupings using the binary outcome yes/no for each pet type at each time point, resulting in pet ownership history variables for each age which can be used to assess pet ownership patterns over time. For example, for dog ownership, clusters were formed for whether participants always, never or sometimes owned a dog or up to age 11, 13, 15 and 18 years.

Potential risk factors and confounding variables (including concurrent ownership of other pets) were examined for association with ownership of each pet type at the earliest time point available for adolescence, which is 13 years. This was deemed a suitable age to compare to childhood pet ownership at age 7, as it was predicted that the ownership of certain pet types is likely to decrease in later adolescence. Socio-demographic variables included gender, ethnicity of the child, number of people in household, presence of an older sibling, maternal and paternal education and social class, maternal age at delivery, whether the mother had pets as a child, and house type (See Table 1). These variables were chosen to match the potential confounders that were used in the childhood models [37]. The variables were entered into multivariable logistic regressions modelling the self-reported ownership of each specific pet type at child age 13 years. A model was not built for tortoises/turtles due to low frequency of ownership of these pets.

**Table 1:** Potential confounders, method and time of data collection, and level of analysis

Variable	Method and time of data collection	Levels
Ownership of a Cat, Dog, Rabbit, Rodent, Bird, Fish, Tortoise/turtles and Horse ownership	Collected retrospectively at 11, 13, 15 and 18 years	No, yes
Gender	Medical records at birth	Male or female
Ethnicity of child	Carer questionnaire at 140 months (11 years)	White, mixed, Asian, black, other. Collapsed to ‘white’ and ‘other’
Number of people in household	Derived from mother’s questionnaire at 122 months (10 years)	3, 4, 5+
Presence of an older sibling	Derived from mother’s questionnaire (child based) at 18 months	No, yes
Maternal education	Mother’s questionnaire at 32 weeks gestation. Highest level indicated	CSE or no qualification (lowest), vocational, O level, A level, degree (highest)
Paternal education	Mother’s questionnaire at 32 weeks gestation. Highest level indicated	CSE or no qualification (lowest), vocational, O level, A level, degree (highest)
Maternal social class	Derived from mother’s questionnaire at 32 weeks gestation (occupation)	Professional (highest), Managerial and technical, Skilled: non-manual, Skilled: manual, Partly skilled, Unskilled (lowest)
Paternal social class	Derived from mother’s questionnaire at 32 weeks gestation (occupation)	Professional (highest), Managerial and technical, Skilled: non-manual, Skilled: manual, Partly skilled, Unskilled (lowest)
Maternal age at delivery	Clinical records	Continuous (years) OR < 21 years, 21–30 years, > 30 years
Mother had pets as a child	Mother’s questionnaire at 33 months	No, not at all; Yes, part of time; Yes, always
House type	Derived from mother’s questionnaire at 122 months (10 years)	Detached, semi-detached, end terrace, terraced, flat/room in someone else’s house/other

To address the problem of partial non-response among confounders, missing data were imputed using multivariate imputation by chained equations (MICE) [50]. These included number of people in household, presence of an older sibling, maternal education, paternal education, maternal social class, paternal social class, maternal age at delivery, mother had pets as a child and house type (detached, semi-detached, end terrace, terraced, flat).

A large difference in sample sizes between ages 13 and 7, even after multiple imputation, made direct comparison of samples challenging because observed differences could result from sample attrition or non-response, rather than age (non-respondents in ALSPAC are likely to differ in terms of socio-economic status [49]). Therefore, inferences from imputed models are not presented. In different approach, a comparison was made by rerunning the age 7 models only for those participants who had provided data at age 13, effectively using the same sample. These complete case models were compared to the original age 7 models (with the exception of horse ownership as this data was not available at age 7). Not all of the predictors identified at 7 years of age were statistically significant at 13 years (with the exception of gender and concurrent pet ownership), although generally speaking, when examining ORs and 95% CIs, trends pointed in the same direction. It is important to note that these predictors of pet ownership may vary due to differences in sample size. Furthermore, a second comparison was made for the 13 year old models; children excluded from the study due to non-response were compared on key characteristics from those who were included in the final sample at age 13 years (Table 3).

Step-wise backwards elimination, using the likelihood ratio, was used to manually remove variables from each model. Variables remained in the model if there was good evidence for an association (*P* < 0.05) or if removal resulted in substantial change to the effect of other variables (10% or greater). As two-way interaction terms between the variable ‘mother owned pets as a child’ and other predictor variables were tested at age 7 [37], this was repeated at age 13, as a reasonable assumption that mother’s pet ownership history may continue to influence adolescent pet ownership. The final models were confirmed with stepwise forward addition. The fit of the model was assessed using the Hosmer-Lemeshow statistic.

## Results

### Pet ownership trends during childhood and adolescence

**Fig. 1:**
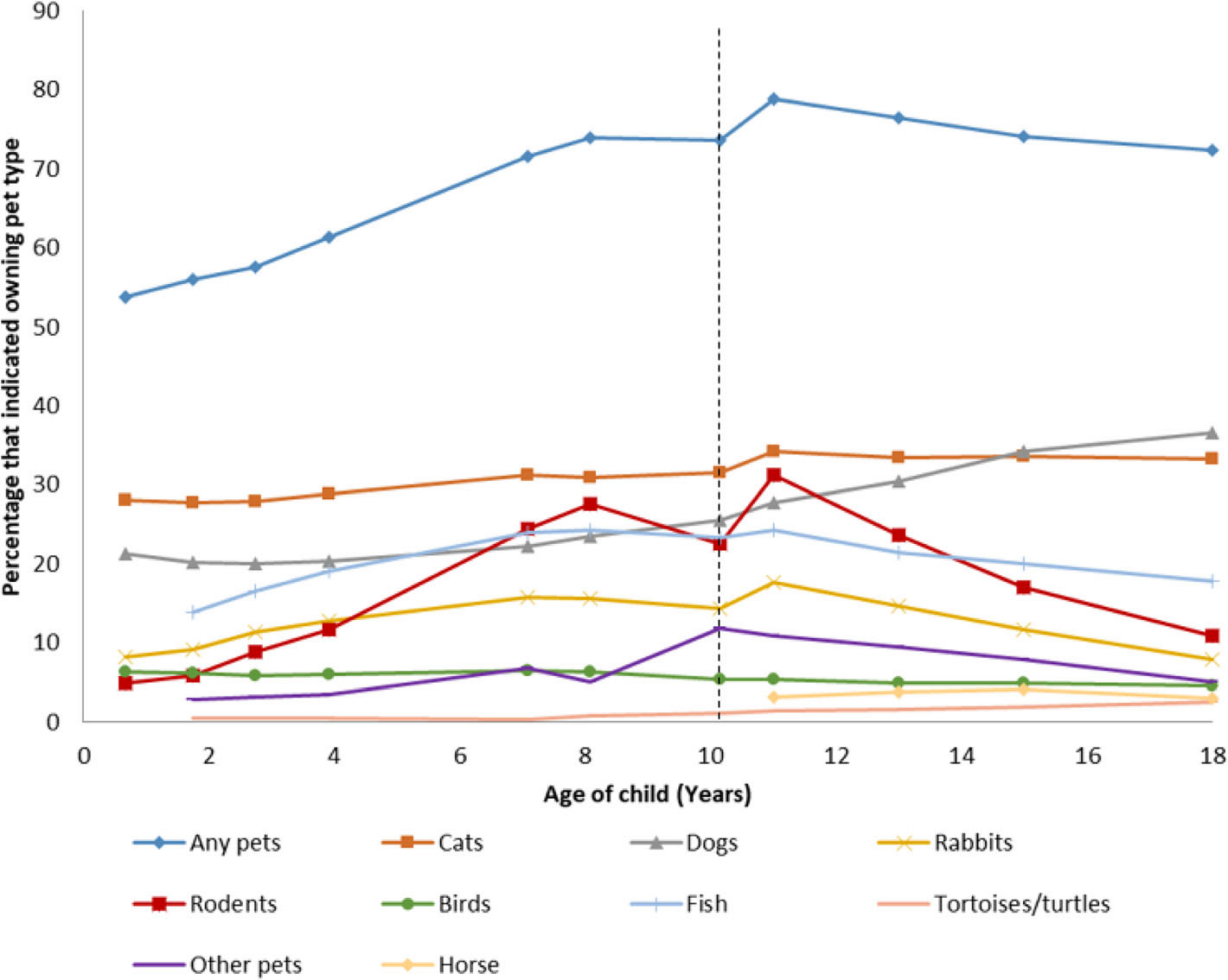
Ownership of different pet types reported in the ALSPAC cohort from 8 months up to age 18 years. Dotted line indicates 10 years; pet ownership data up to 10 years were caregiver-reported and published previously [37]. Pet ownership for ages 11–15 years was self-reported by youth at 18 years

During gestation, 58% of mothers reported owning a pet. Family pet ownership of all types changed across childhood and adolescence (Fig. 1). By age 10 years, pet ownership had risen to 74%, cat ownership was 31% and dog ownership was 26%. There was an increase over time in the frequency of ownership of fish, rodents and rabbits until age 11 years. Thereafter, pet ownership of all pet types other than cats and dogs declined. By age 18 years, pet ownership stayed reasonably constant at 72%, and dog ownership had risen to 37%. Cats were the most commonly reported pet up to age 15 years; dogs were the most common pet type among older adolescents. This is not consistent with nationwide data, where cat and dog ownership was reported to be equal from 2008 to 2012 [51].

**Fig. 2:**
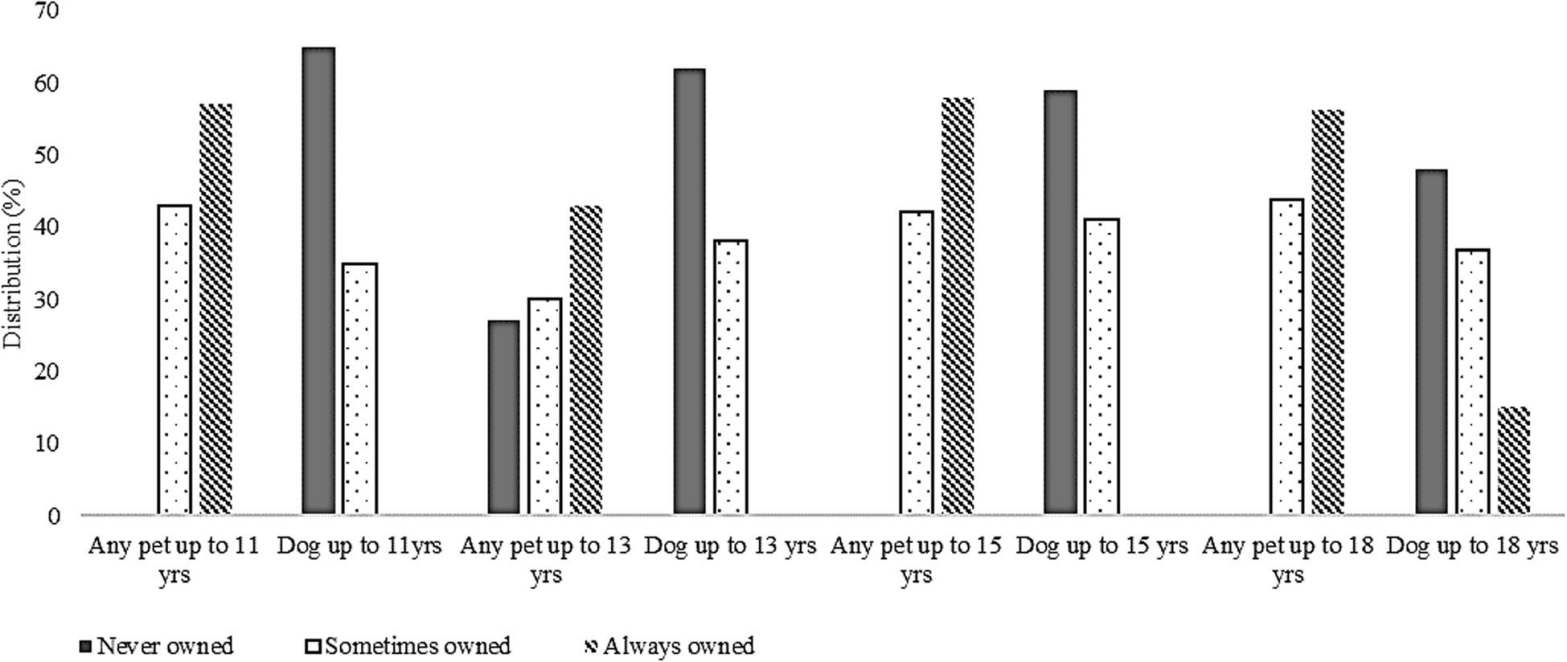
Two-step cluster analysis in SPSS to identify ownership length of pet-ownership types

Using two-step cluster analysis, clusters emerged from pet ownership up to ages 11, 13, 15 and 18. Including data up to age 11, age 15 and 18 years, only two pet ownership clusters were identified, subsequently termed: sometimes owned a pet; and always owned a pet (Fig. 2). When considering data from all years up to age 13, three pet ownership clusters were identified: never owned a pet; sometimes owned a pet; and always owned a pet (Fig. 2).

There is an increased interest in researching the health benefits of dog ownership, perhaps due to a higher level of interaction and reciprocation in comparison to other pets. Therefore process was repeated for history of dog ownership. Dog ownership up to 11, 13 and 15 years formed two clusters: never owned a dog; and sometimes owned a dog (Fig. 2). Dog ownership up to 18 years formed 3 clusters: never owned a dog; sometimes owned a dog; and always owned a dog (Fig. 2).

**Fig. 3:**
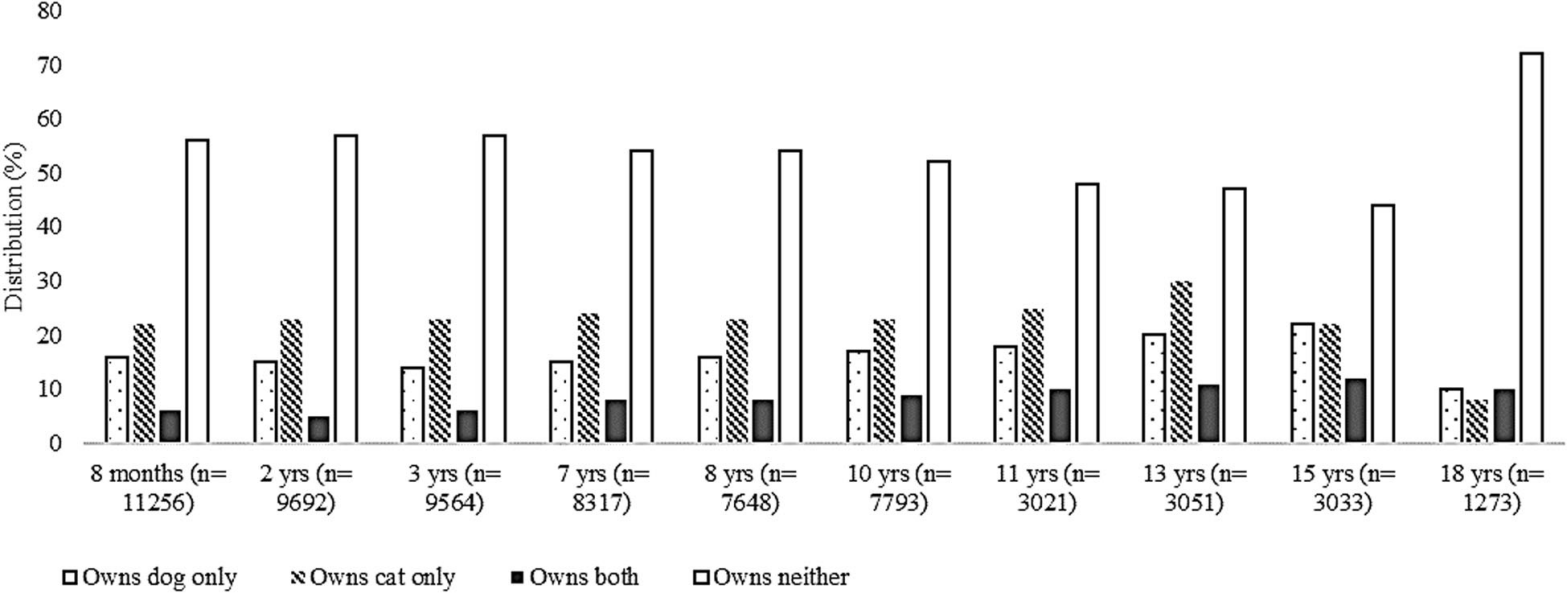
Two-step cluster analysis in SPSS to segregate reported dog-ownership from cat-ownership

Because cat and dog ownership was the most frequently reported, using two-step cluster analysis, further clusters were identified at each age for: own dog only; owns cat only; owns both dog and cat; owns neither dog nor cat (Fig. 3). With the use of these clusters, it will be possible to separate out the effects of dog and cat ownership in future research.

### Characteristics of sample at 13 years old

A comparison for the characteristics of the study children with reported pet ownership status at ages 7 and 13 years are described in Table 2. At age 13, a higher proportion of the sample are female, and have a higher maternal and paternal education in comparison to age 7 (Table 2). The characteristics of the study children at age 13 years are compared to the excluded children with no pet ownership data at age 13 years in Table 3. The excluded sample were more likely to be male, with a lower maternal and paternal education (Table 3).

**Table 2:** Characteristics of the study children at ages 7 and 13 years with a reported ownership of any pets

Variable	Level	Age 7 (*n* = 8331)	Age 13 (*n* = 2332)
		Number (%)	Number (%)
Gender	Male	4312 (52)	751 (32)
	Female	4019 (48)	1580 (68)
Ethnicity	White	6068 (97)	1868 (97)
	Non-white	422 (3)	50 (3)
Number of people in household	3	1233 (15)	323 (15)
	4	4168 (50)	1138 (51)
	5+	2904 (35)	774 (34)
Presence of an older sibling at 18 months	Yes	4323 (54)	1140 (51)
	No	3636 (46)	1095 (49)
Maternal education	CSE or no qualification (lowest)	1631 (21)	222 (10)
	Vocational	710 (9)	166 (7)
	O level	2873 (35)	722 (32)
	A level	2102 (26)	647 (29)
	Degree (highest)	1269 (16)	478 (21)
Paternal education	CSE or no qualification (lowest)	1631 (21)	366 (16)
	Vocational	639 (8)	165 (7)
	O level	1711 (22)	451 (20)
	A level	2199 (28)	645 (29)
	Degree (highest)	1683 (21)	608 (27)
Maternal social class	Professional (highest)	478 (7)	189 (9)
	Managerial and technical	2365 (34)	814 (36)
	Skilled: non-manual	2957 (43)	892 (40)
	Skilled: manual	467 (7)	131 (6)
	Partly skilled	550 (8)	176 (8)
	Unskilled (lowest)	116 (2)	33 (2)
Paternal social class	Professional (highest)	941 (13)	344 (15)
	Managerial and technical	2667 (36)	839 (38)
	Skilled: non-manual	858 (12)	274 (12)
	Skilled: manual	2154 (29)	574 (26)
	Partly skilled	603 (8)	167 (8)
	Unskilled (lowest)	189 (3)	37 (2)
Maternal age at delivery	< 21 years	303 (4)	56 (3)
	21–30 years	5043 (61)	1292 (58)
	> 30 years	2985 (36)	887 (40)
Mother had pets as a child	No, not at all	743 (10)	196 (9)
	Yes, part of time	3517 (46)	994 (45)
	Yes, always	3365 (44)	1045 (47)
House type	Detached	2443 (29)	764 (34)
	Semi-detached	3086 (27)	801 (36)
	End terrace	771 (9)	198 (9)
	Terraced	1652 (20)	396 (18)
	Flat/room in someone else’s house/other	336 (4)	76 (3)

**Table 3:** Characteristics of the study children at age 13 years in comparison to excluded children at age 13 years (with no pet ownership data due to non-response)

Variable	Level	Excluded Study Children (*n* = 4120)	Included study children (*n* = 2332)
		Number (%)	Number (%)
Gender	Male	2383 (58)	751 (32)
	Female	1737 (42)	1580 (68)
Ethnicity	White	2710 (97)	1868 (97)
	Non-white	78 (3)	50 (3)
Number of people in household	3	487 (15)	323 (15)
	4	1525 (48)	1138 (51)
	5+	1149 (36)	774 (34)
Presence of an older sibling at 18 months	Yes	2326 (59)	1140 (51)
	No	1599 (41)	1095 (49)
Maternal education	CSE or no qualification (lowest)	675 (17)	222 (10)
	Vocational	412 (10)	166 (7)
	O level	1484 (37)	722 (32)
	A level	988 (25)	647 (29)
	Degree (highest)	429 (11)	478 (21)
Paternal education	CSE or no qualification (lowest)	912 (24)	366 (16)
	Vocational	372 (10)	165 (7)
	O level	907 (24)	451 (20)
	A level	1074 (28)	645 (29)
	Degree (highest)	584 (15)	608 (27)
Maternal social class	Professional (highest)	167 (5)	189 (9)
	Managerial and technical	1069 (32)	814 (36)
	Skilled: non-manual	1490 (44)	892 (40)
	Skilled: manual	250 (7)	131 (6)
	Partly skilled	313 (9)	176 (8)
	Unskilled (lowest)	73 (2)	33 (2)
Paternal social class	Professional (highest)	350 (10)	344 (15)
	Managerial and technical	1202 (33)	839 (38)
	Skilled: non-manual	389 (11)	274 (12)
	Skilled: manual	1211 (34)	574 (26)
	Partly skilled	339 (9)	167 (8)
	Unskilled (lowest)	120 (3)	37 (2)
Maternal age at delivery	< 21 years	174 (4)	56 (3)
	21–30 years	2596 (63)	1292 (58)
	> 30 years	1350 (33)	887 (40)
Mother had pets as a child	No, not at all	256 (7)	196 (9)
	Yes, part of time	1574 (43)	994 (45)
	Yes, always	1862 (50)	1045 (47)
House type	Detached	1073 (26)	764 (34)
	Semi-detached	1586 (39)	801 (36)
	End terrace	403 (10)	198 (9)
	Terraced	871 (21)	396 (18)
	Flat/room in someone else’s house/other	170 (4)	76 (3)

### Multivariable models for age 13 data

The results presented in the tables are data derived from multiple imputation. Complete-case analyses for all models were identical.

#### Cat ownership

The final multivariable model of cat ownership at 13 years is presented in Table 4, alongside univariable data for comparison. Participants were more likely to own a cat if they owned fish, more likely if they were female and if maternal age at delivery was older (> 30 years). Participants with maternal pet ownership history (sometimes or always) were more likely to own a cat compared to children whose mothers did not own pets during childhood. The Hosmer-Lemeshow statistic was high (0.77), suggesting good model fit.

**Table 4:** Multivariable binary logistic regression model of cat ownership at 13 years among children who reported any pet ownership

Variable	Univariable result (unadjusted)			Final adjusted model		
	OR	95%CI	P	OR	95%CI	P
Fish						
No	1			1		
Yes	1.45	1.21–1.73	< 0.001	1.40	1.16–1.69	< 0.001
Gender						
Male	1			1		
Female	1.28	1.08–1.49	0.003	1.29	1.09–1.53	0.003
Maternal age at delivery						
< 21 yrs	1		0.155	1		0.008
21–30 yrs	1.31	0.78–2.18	0.305	1.44	0.85–2.44	0.164
> 30 yrs	1.47	0.88–2.46	0.141	1.79	1.05–3.04	0.030
Mother had pets as a child						
No, not at all	1		< 0.001	1		< 0.001
Yes, part of the time	1.55	1.08–2.23	0.017	1.56	1.09–2.25	0.015
Yes, always	3.08	2.19–4.30	< 0.001	3.10	2.21–4.37	< 0.001

Hosmer-Lemeshow statistic = 0.77, *n* = 2923

#### Dog ownership

The final multivariable model of dog ownership at 13 years is presented in Table 5, alongside univariable results for comparison. Participants were more likely to own a dog if they also owned a bird, fish or horse. Participants with an older sibling were more likely to report owning a dog. The older the mother was at delivery, the less likely the child was to report living with a dog. The Hosmer-Lemeshow statistic was high (0.83), suggesting good model fit.

**Table 5:** Multivariable binary logistic regression model of dog ownership at 13 years

Variable	Univariable result (unadjusted)			Final adjusted model		
	OR	95%CI	P	OR	95%CI	P
Bird						
No	1			1		
Yes	2.53	1.81–3.52	< 0.001	2.12	1.47–3.03	< 0.001
Fish						
No	1			1		
Yes	1.43	1.19–1.71	< 0.001	1.29	1.06–1.57	0.009
Horse						
No	1			1		
Yes	10.32	6.43–16.55	< 0.001	10.43	6.34–17.18	< 0.001
Older sibling at 18 months						
No	1			1		
Yes	1.36	1.15–1.59	< 0.001	1.50	1.26–1.79	< 0.001
Maternal age at delivery						
< 21 yrs	1		< 0.001	1		< 0.001
21–30 yrs	0.46	0.29–0.73	0.001	0.44	0.27–0.72	< 0.001
> 30 yrs	0.36	0.23–0.58	< 0.001	0.32	0.20–0.53	< 0.001

Hosmer-Lemeshow statistic = 0.83, *n* = 2922

#### Rabbit ownership

The Hosmer-Lemeshow statistic for the rabbit model was low (0.22), suggesting a poor model fit. It is difficult to determine why the model was a poor fit, we suggest it could be due to additional unknown confounding variables which have not been included in the model. The final multivariable model of rabbit ownership at 13 years is presented in Table 6, alongside univariable results for comparison. Participants were more likely to report owning a rabbit if they also owned a rodent, fish, horse and were female. Those with maternal education at degree level were less likely to own a rabbit. Participants who had mothers who sometimes and always owned pets as a child were also more likely to own a rabbit than if their mothers never owned pets as a child.

**Table 6:** Multivariable binary logistic regression model of rabbit ownership at 13 years

Variable	Univariable result (unadjusted)			Final adjusted model		
	OR	95%CI	P	OR	95%CI	P
Rodent						
No	1			1		
Yes	2.23	1.80–2.77	< 0.001	1.98	1.58–2.48	< 0.001
Fish						
No	1			1		
Yes	1.82	1.46–2.28	< 0.001	1.60	1.26–2.02	< 0.001
Horse						
No	1			1		
Yes	2.20	1.43–3.39	< 0.001	1.92	1.22–3.01	0.005
Gender						
Male	1			1		
Female	1.69	1.35–2.12	< 0.001	1.53	1.21–1.94	< 0.001
Maternal Education						
CSE/None	1		0.002	1		0.014
Vocational	0.59	0.35–1.04	0.052	0.61	0.35–1.04	0.68
O Level	0.90	0.64–1.28	0.582	0.94	0.66–1.35	0.750
A Level	0.86	0.60–1.23	0.393	0.89	0.62–1.28	0.544
Degree	0.53	0.35–0.78	0.001	0.56	0.37–0.84	0.005
Mother had pets as a child						
No, not at all	1		0.004	1		0.023
Yes, part of the time	1.71	1.10–2.64	0.016	1.70	1.09–2.64	0.019
Yes, always	1.96	1.27–3.03	0.002	1.70	1.109–2.64	0.018

Hosmer-Lemeshow statistic = 0.22, *n* = 2656

#### Rodent ownership

The final multivariable model of rodent ownership at 13 years is presented in Table 7, alongside univariable results for comparison. Participants were more likely to report owning a rodent if they: owned a rabbit, fish, were female, had higher numbers of people living in the household, their mother sometimes owned pets as a child. Participants were less likely to report owning a rodent if they had older siblings and a lower maternal education. The Hosmer-Lemeshow statistic was high (0.92), suggesting good model fit.

**Table 7:** Multivariable binary logistic regression model of rodent ownership at 13 years

Variable	Univariable result (unadjusted)			Final adjusted model		
	OR	95%CI	P	OR	95%CI	P
Rabbit						
No	1			1		
Yes	2.23	1.80–2.77	< 0.001	1.78	1.39–2.31	< 0.001
Fish						
No	1			1		
Yes	1.92	1.58–2.32	< 0.001	1.94	1.56–2.42	< 0.001
Gender						
Male	1			1		
Female	2.01	1.66–2.43	< 0.001	2.12	1.79–2.63	< 0.001
Number of people in household						
3	1		0.005	1		0.018
4	1.37	1.04–1.83	0.028	1.42	1.04–1.95	0.027
5+	1.65	1.22–2.26	0.002	1.62	1.16–2.26	0.005
Older sibling at 18 months						
No	1			1		
Yes	0.912	0.76–1.09	0.305	0.75	0.66–0.97	0.005
Maternal education						
CSE/None	1		0.29	1		0.037
Vocational	0.70	0.46–1.08	0.113	0.57	0.46–1.13	0.035
O Level	0.75	0.55–1.01	0.056	0.59	0.56–1.05	0.004
A Level	0.85	0.63–1.16	0.312	0.71	0.66–1.26	0.56
Degree	0.87	0.43–1.19	0.403	0.77	0.73–1.41	0.163
Mother pets as a child						
No, not at all	1		0.001	1		0.029
Yes, part of time	1.04	0.76–1.44	0.792	1.03	0.73–1.44	0.013
Yes, always	1.44	1.04–2.01	0.030	1.37	0.97–1.94	0.742

Hosmer-Lemeshow statistic = 0.92, *n* = 2863

#### Bird ownership

The final multivariable model of bird ownership at 13 years is presented in Table 8, alongside univariable results for comparison. Participants were more likely to have a bird if they also owned a fish or horse. Likelihood of owning a bird decreased with increasing maternal education level, and was highest in skilled manual, and part-skilled paternal occupations.

**Table 8:** Multivariable binary logistic regression model of bird ownership at 13 years

Variable	Univariable result (unadjusted)			Final adjusted model		
	OR	95%CI	P	OR	95%CI	P
Fish						
No	1			1		
Yes	2.34	1.66–3.30	< 0.001	2.29	1.60–3.28	< 0.001
Horse						
No	1			1		
Yes	3.79	2.19–6.54	< 0.001	3.68	2.07–6.53	< 0.001
Maternal education						
CSE/None	1		< 0.001	1		0.006
Vocational	0.41	0.18–0.92	0.031	0.39	0.16–0.90	0.028
O Level	0.50	0.31–0.81	0.005	0.54	0.32–0.86	0.016
A Level	0.48	0.29–0.80	0.005	0.632	0.37–1.07	0.09
Degree	0.17	0.08–0.35	< 0.001	0.26	0.12–0.55	0.001
Paternal Social Class						
Professional	1		< 0.001	1		0.003
Managerial and technical	1.16	0.58–2.29	0.674	0.96	0.48–1.94	0.899
skilled non-manual	1.12	0.49–2.57	0.785	0.86	0.37–1.99	0.730
skilled manual	2.65	1.42–4.94	0.002	1.92	1.01–4.03	0.060
part skilled	3.92	1.91–8.07	< 0.001	2.72	1.23–5.87	0.010
unskilled	2.45	0.57–10.54	0.222	1.40	0.31–5.64	0.66

Hosmer-Lemeshow statistic = 0.57 *n* = 2922

#### Fish ownership

Model is not presented as according to the Hosmer-Lemeshow statistic (0.005), it was not a good fit for the data.

#### Horse ownership

The final multivariable model of horse ownership at 13 years is presented in Table 9, alongside univariable results for comparison. Participants were more likely to own a horse if they owned a dog, rabbit, or were female. Participants living in a semi-detached and terraced house were less likely to own a horse (in comparison to living in a detached house). The Hosmer-Lemeshow statistic was very high, (0.92) suggesting good model fit.

**Table 9:** Multivariable binary logistic regression model of horse ownership at 13 years

Variable	Univariable result (unadjusted)			Final adjusted model		
	OR	95%CI	P	OR	95%CI	P
Dog						
No	1			1		
Yes	10.32	6.43–16.55	< 0.001	10.43	6.36–17.10	< 0.001
Rabbit						
No	1			1		
Yes	2.20	1.43–3.39	< 0.001	1.37	0.79–2.37	0.006
Gender						
Male	1			1		
Female	3.01	1.81–.5.02	< 0.001	3.15	1.82–5.45	< 0.001
House Type						
Detached	1		0.002	1		0.004
Semi-detached	0.57	0.36–0.91	0.019	0.57	0.35–0.94	0.027
End terrace	0.62	0.29–1.32	0.214	0.62	0.28–1.35	0.235
Terraced	0.32	0.15–0.66	.002	0.33	0.15–0.69	0.003

Hosmer-Lemeshow statistic = 0.92 *n* = 2866

## Discussion

This paper describes patterns of pet ownership data in the ALSPAC cohort from 11 to 18 years, and presents multivariable models of pet ownership at 13 years of age to determine what confounding factors are important to take into account in future HAI studies. Consistent with the childhood findings, we find similar factors contributing to the ownership of different pet types in adolescence. Interestingly, the interaction effects observed in childhood [37] were not present in the adolescent data, particularly the interaction between maternal pet ownership in childhood and maternal or paternal education in regard to cat and dog ownership.

It was previously observed that family pet ownership increased during childhood (up to age 10 years) [37], and was expected to continue on this trajectory. However, in the present study, pet ownership peaked at age 11 for all pet types, then slightly decreased afterwards for all pet types except cats and dogs, which slightly increased. The largest decrease was in the ownership of small pets (rabbits, fish and rodents) which likely explains the descent in pet ownership as a whole in adolescence. All other pet types stayed fairly constant. These findings are consistent with reports on pet-ownership among adolescents in Great Britain [43].

Our findings are similar to research suggesting that marginally higher levels of pet ownership exist in middle childhood (between 8 and 12-years-old) [39, 52, 53] compared to infancy and adolescence. In ALSPAC, cat ownership remained reasonably constant from ages 11 to 18 years; dog ownership increased and overtook cats as the most common pet. This is consistent with other data from UK [36, 38, 54, 55] and English, Scottish, and Welsh households [36, 42, 56]. Other research has also found small mammal ownership to decrease, but dog ownership to increase throughout adolescence [43]. In a study examining the socio-demographics of pet ownership among adolescents in Great Britain [43], 15-year-old (OR = 1.146, *p* < 0.001) and 13-year-old (OR = 1.240, *p* = 0.021) adolescents were significantly more likely than 11-year-old adolescents to own dogs, and less likely to own fish, reptiles, or amphibians (OR = 0.629, *p* < 0.001) and small mammals (OR = 0.630, *p* < 0.001). Interestingly, in ALSPAC dog ownership did not follow a linear trend across childhood; in infancy and young childhood, dog ownership declined, suggesting families were more likely to acquire a dog once the youngest child in the family reaches middle childhood. This supports findings that dogs are more common in households with older children [38].

Among ALSPAC children, owning one type of small pet was commonly associated with owning another type. However, no evidence was found for an association with dog ownership and cat ownership, and vice versa, similar to childhood [37]. This is consistent with other null findings on joint cat and dog ownership [55], but is at odds with studies in the UK and Ireland that do find associations [34, 36, 57]. Dog ownership among ALSPAC children at age 13 years reflected the findings from 7 years [37] in terms of concurrent bird and fish ownership. At age 13, those who owned a horse were also more likely to own a dog. This finding is consistent with observations from other studies [38]. However, in ALSPAC we cannot discuss about trends with earlier ages because at age 7, horse ownership was not queried as a separate pet category.

In contrast to findings at 7 years, there was no relationship between cat or dog ownership and education level of the mother or the father at age 13. This also contrasts with other studies which showed that cat owners had higher [36] and lower [41] education levels than those without cats, and that dog ownership decreased as owners’ education increased [34, 36, 41]. According to other research, adolescents were more likely to report having pet dogs if their parents were employed [43], while those with a medium family affluence level were less likely to own a cat than those with a low family affluence level [43]. Associations between dog ownership and lower social class [34] or family affluence [43] have been previously reported. The present study did not find any association between dog ownership and paternal or maternal social class at adolescence, as it had for childhood [37]. Rabbit, rodent and bird ownership models at age 13 identified similar predictors as those obtained at age 7, in terms of education and social class. Likelihood of owning a bird decreased with higher maternal education, and was highest in skilled manual, and part-skilled paternal occupations at both ages. In a previous study, adolescents were less likely to own birds if their family had a medium or higher family affluence level than adolescents with low family affluence level [43].

Previous pet ownership is related to current and future pet ownership [58, 59]. At age 7 years, bird ownership was the only pet type not affected by whether the mother owned pets as a child; this changed at age 13 years, where both dog ownership and horse ownership were also not affected by whether the mother owned pets as a child. Horse ownership at age 13 cannot be compared to ownership at age 7 years, as it was not measured on previous occasions. The finding that at adolescence dog ownership was not explained by mothers’ previous pet ownership could be due to greater participation of the adolescent in the decision to obtain a pet, thus reducing the influence of maternal pet ownership history. However, for horse ownership it can be argued that financial considerations may depend on the parent, and therefore the decision to obtain a horse is likely more complex. At age 13 years, maternal pet ownership did predict rabbit ownership, as had been the case at age 7 years. One limitation of our data is that we do not know the individual pet types the mother had owned as a child, and no qualitative data was collected on reasoning to own a particular type of pet, therefore, any interpretation relies on speculation.

Findings differed slightly between the 7 and 13-year models in terms of maternal age at delivery. At age 7, maternal age at delivery was independently associated with dog and rabbit ownership, with likelihood of ownership decreasing as maternal age increased. At age 13 years, maternal age at delivery was inversely associated with the likelihood of dog ownership, whereas it was positively associated with cat ownership. Westgarth et al. (2010) [37] concluded these associations were not likely due to socio-economic differences between mothers who give birth when they are older or younger, as socio-economic differences were also included in the model.

In the present study, the number of people in the household was only positively associated with rodent ownership at age 13. Larger household size has also been associated with dog ownership in the UK [36, 38], and in ALSPAC children [37], but not in other studies [57]. Previous research also suggests that larger families are more likely to have companion animals [43, 60]. Why this differs at adolescence is not clear, but may be due to the difference in sample size. Research regarding pet ownership and numbers of siblings is inconsistent [19, 39, 42, 44, 46, 61].

In addition to family size, being the youngest sibling may be an explanatory factor. At age 7 years, the presence of an older sibling was an independent predictor of family ownership of dogs, rodents, birds and fish. At age 13 years, there is only evidence of an association between the presence of older siblings and the likelihood of dog or rodent ownership. Other research has suggested that youths with younger siblings own fewer pets than those without younger or any siblings [39]. However, the ALSPAC findings are difficult to dissect because, overall dog ownership increases whereas rodent ownership declines across adolescence, and yet their association with sibling age is similar.

The associations with gender and pet type at age 13 years were identical to the models at age 7; females were more likely to own cats, rabbits, and rodents. In addition, females were more likely to own horses. These findings are consistent with other studies on cats [34, 36, 42, 57], rodents and horses [42]; still other studies found no gender differences [24, 43, 44]. It has been suggested that girls may influence their parents to own certain types of pets [37]. We have difficulty inferring the influence of gender on family pet ownership, as family structures are likely to have both sexes [35, 39, 43], and more than one attribute of the child and/or the family affects the decision to get a pet.

At age 13 years, house type was only associated with horse ownership. This is at difference with the models at age 7 years where house type was associated with both dog and rabbit ownership. Westgarth et al. [37] suggested this could be explained by family reasoning that dogs and rabbits are perceived to require more outdoor space than other pet types, which could also explain the reasoning for horses. Although maternal education and social class were not significant in the final model, socio-economic status (SES) should not be discarded as a potential influence for horse ownership as house type is a measure of SES.

At age 7 years, ethnicity other than ‘white’ was associated with a lower likelihood of owning a cat or rodent [37]. However, at age 13 years, ethnicity is not related to any pet type. This is at odds with finding that adolescents were more likely to report owning cats if they were white compared with non-white adolescents [43]. Other research finds ethnicity to be the single most important predictor of pet ownership, with white adolescents being more likely to own any types of pets than non-white adolescents (Mixed, Asian, Black, and adolescents from other ethnicities) [43]. This is supported by other studies in adolescents [44] and young adults [62].

The lack of association in the ALSPAC cohort at age 13 years may be due to insufficient power. In the ALSPAC dataset the prevalence of ethnic minorities is relatively low [49].

This study has some limitations. First, the accuracy of retrospective recall of pet ownership could be questioned. However, recall accuracy has been tested for age 7, when it was compared to data provided prospectively by caregivers on previous occasions. We found a high level of consistency between caregiver-reported and youth-recalled pet ownership (*P* < 0.0001). Secondly, there may be other confounding variables that were not considered in the models. Other potential confounders could be considered, for example measures of family adversity. The present findings cannot be generalised to all populations of children and adolescents in the UK. Although the cohort was broadly representative of UK populations at baseline, attrition of participants over time lead to certain differences, for example in ethnicity and social class [49]. There were marked differences between excluded and included study children at age 13; non-response participants were more likely to be male and from a lower socio-economic background. It is important to note this difference when comparing findings to other UK pet ownership studies, or further afield. However, advantages of the ALSPAC dataset are numerous, and include a large sample size, longitudinal data collection, and availability of a wide range of confounding factors for multivariable analysis.

## Conclusions

Many children grow up with pets, therefore it is important to investigate any potential psychological and physical benefits of pet ownership to child health. Due to limitations in study design and data analysis of research published to date [11], it has been difficult to determine whether any of the associations reported could be explained by residual confounding. Using the ALSPAC birth cohort, we showed that in adolescence, a number of socio-economic and demographic factors are associated with the ownership of different pet types. Therefore, the relevant factors to specific pet types must be accounted for in data analysis of pet ownership and improved health outcomes. In our analyses, maternal age at delivery, maternal education, and family structure were commonly reported indicators of pet ownership, and are likely to have independent effects on child health and development. These factors are potential confounders in public health research and must be accounted for in future HAI studies.

## Data Availability

The data that support the findings of this study are available from ALSPAC but restrictions apply to the availability of these data, which were used under license for the current study, and so are not publicly available. Data are however available from the authors upon reasonable request and with permission of ALSPAC.

## Abbreviations

ALSPAC: Avon Longitudinal Study of Parents and Children
HAI: Human-Animal Interaction
MICE: Multiple Imputation of Chained Equations
